# Peer punishment promotes enforcement of bad social norms

**DOI:** 10.1038/s41467-017-00731-0

**Published:** 2017-09-20

**Authors:** Klaus Abbink, Lata Gangadharan, Toby Handfield, John Thrasher

**Affiliations:** 1 Department of Economics, Monash University, Melbourne, VIC 3800 Australia; 2 SOPHIS, Faculty of Arts, Monash University, Melbourne, VIC 3800 Australia

## Abstract

Social norms are an important element in explaining how humans achieve very high levels of cooperative activity. It is widely observed that, when norms can be enforced by peer punishment, groups are able to resolve social dilemmas in prosocial, cooperative ways. Here we show that punishment can also encourage participation in destructive behaviours that are harmful to group welfare, and that this phenomenon is mediated by a social norm. In a variation of a public goods game, in which the return to investment is negative for both group and individual, we find that the opportunity to punish led to higher levels of contribution, thereby harming collective payoffs. A second experiment confirmed that, independently of whether punishment is available, a majority of subjects regard the efficient behaviour of non-contribution as socially inappropriate. The results show that simply providing a punishment opportunity does not guarantee that punishment will be used for socially beneficial ends, because the social norms that influence punishment behaviour may themselves be destructive.

## Abstract (web summary)

Punishment by peers can enforce social norms, such as contributing to a public good. Here, Abbink and colleagues show that individuals will enforce norms even when contributions reduce the net benefit of the group, resulting in the maintenance of wasteful contributions.

## Introduction

Moral, social and legal norms are crucial in sustaining the very high level of cooperation with non-relatives that is observed in human societies. Norms involve a commitment to behave in conformity with a rule, conditional on sufficiently many others sharing the commitment to that rule. They also may involve a commitment among some norm followers to punish transgressions of the norm^1–4^. Punishment is likely to be especially important for the maintenance of norms that arise in social dilemmas, where there are conflicts between individual and collective interests. Economics experiments using public goods games show that providing subjects with the opportunity to inflict punishment in a social dilemma promotes higher levels of cooperation^1^. Although the losses created by punishment sometimes outweigh the gains of cooperation^1, 5–7^, coordination of punishment^8^ and also longer periods of repeated interaction^1, 9^ make it very likely to be that cooperation will spread and benefit the group overall. In these cases, punishment is likely to be used to promote norms of fairness, which have prosocial effects. Punishment is used to increase social welfare even at a personal cost to the punisher; hence, the term ‘altruistic punishment’ has been coined to describe these phenomena^1^.

Not all norms, however, are socially beneficial. For example, in some cultures, norms of sexual purity motivate punishment of transgressions, including so-called ‘honour killings’ of rape victims^10–12^. Cultures of honour subscribe to norms that require violent retaliation for trivial slights, often leading to devastating escalations of violence^13^. Even apparently benign norms of gift-giving have been identified as responsible for costly inefficiencies, amounting to billions of dollars in annual deadweight loss^14^.

This study aims to investigate whether punishment will be employed to establish socially costly norms in a paradigm that resembles earlier public goods experiments. In typical public goods games, some subjects appear to hold normative attitudes that require positive, equal contributions from all members. These attitudes are likely to combine elements of fairness (‘we should all contribute equally’) and benevolence (‘by contributing I/we make others better off’). We vary the standard public goods design, however, by setting the social benefit from contributing to be zero or negative. That is, although one individual’s contributions will benefit the remainder of the group, they do not make the group better off as a whole. In this setting, normative attitudes that require positive contributions will be potentially harmful to the collective. We hypothesise that providing subjects with the opportunity to punish in such games will allow these potentially damaging norms to influence behaviour much more than they would in the absence of punishment. Thus, groups provided with punishment opportunities will contribute more and this outcome will be mediated by the normative attitudes, held by at least some subjects, requiring positive contributions.

As existing studies have used a paradigm in which cooperation was beneficial, it is unknown whether punishment can be used to elicit destructive behaviour. On the one hand, it has been suggested theoretically that if punishment is sufficiently potent, it can institute any norm, no matter how foolish^15^. On the other hand, it is also thought that psychological propensities to adopt and enforce norms have been subject to significant evolutionary pressures, suggesting there may be significant limitations on the range of possible norms^16^. Although some existing evidence suggests that ‘altruistic’ punishment has a dark side^6, 17–20^, our study is the first to provide experimental evidence that subjects will enforce a destructive norm with punishment.

In a (linear) public good game, players are endowed with a number of monetary units (MU), which they can either keep or invest. Invested monies are multiplied by a factor called the marginal per-capita return (MPCR) and every player in the group receives the multiplied amount. If the MPCR is between ^1^/_*n*_ and 1, with *n* being the number of players, there is a social dilemma, where the individual dominant strategy is to keep all one’s endowment, while social welfare is maximised if everybody invests all. This is the environment where punishment has proven to be effective to enforce cooperation in previous experiments.

In this study we remove the social dilemma aspect from the game. In two treatments (P25 and P20) we set the MPCR to 0.25 and 0.2, respectively, where *n* = 4. In the former, there is no welfare gain from investments; in the latter, investments actually lower the group payoff: every dollar contributed leads to a payoff of $0.80, divided equally between the four members. In two baseline treatments (N25 and N20), subjects play the same games, but without a punishment opportunity.

We find that punishment significantly increases contributions, in both the neutral and the destructive environments. A second experiment demonstrates that subjects have shared attitudes that it is appropriate to contribute more than five units and inappropriate to contribute zero. This finding suggests that punishment in the first experiment is being used to enforce a social norm, even though in the present environment the effect of that norm is harmful, or at best neutral, with respect to group payoffs.

## Results

### Contributions and punishment

Eighty-three out of 116 subjects (72%) in punishment treatments punished at least once. As hypothesised, contributions were higher in the punishment treatments than in the controls. In P25, the average contribution per subject, per round was 5.6 MU; in N25, without punishment, it was 1.6. In P20, the average contribution per subject, per round was 3.4; in N20, without punishment, it was 0.7. Both differences are statistically significant (one-sided, Fisher’s two-sample randomisation test^21^, P25 vs. N25: *p* = 0.002, P20 vs. N20: *p* = 0.007).

We report in Table 1 average punishments dispensed and received by subjects who contributed more or less than the mean amount contributed on a given round by their three co-players. Consistent with earlier findings, most punishment is dispensed by high contributors and most punishment is received by low contributors^1, 22^. A regression model, described in Supplementary Table 1, supports this result.

**Table 1:** Average amounts of punishment per round dispensed and received by high and low contributors

	Above average contributors	Below average contributors
Punishment dispensed	1.17 (0.08)	0.47 (0.05)
Punishment received	0.35 (0.04)	1.20 (0.06)

Standard errors in parentheses

**Fig. 1:**
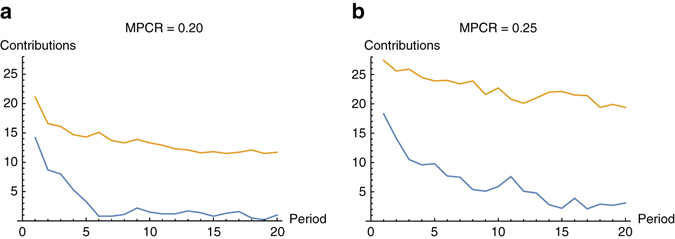
Time series of mean aggregate group contributions per round. **a** MPCR = 0.2, P20 treatment with punishment (*orange*), N20 without punishment (*blue*); **b** MPCR = 0.25, P25 treatment with punishment (*orange*), N25 without punishment (*blue*). (N20, *n* = 15, P20, *n* = 15; N25, *n* = 14; P25, *n* = 14)

In Fig. 1, we report time series of contributions for each of the four treatments. Contributions start out high, but in the non-punishment treatments they decay significantly. Similar to other public goods experiments, we find that punishment stabilises contributions at a significantly higher level^22, 23^. We also observe, consistent with earlier findings^23^, that higher rates of return lead to higher contributions (Fisher’s two-sample randomisation test, one-tailed *p* = 0.066 across the treatments without punishment, *p* = 0.071 across the treatments with punishment). Attempts to determine whether the mere threat of punishment was sufficient to maintain high contributions were inconclusive (Supplementary Fig. 1).

### Earnings

Contributions were destructive in the MPCR = 0.2 treatments and so because contributions were higher in P20 than in N20, group earnings were lower. Net of any expenditure on punishment, subjects in the punishment treatment earned 10.8 MU less on average over the course of the experiment.

### Normative attitudes

**Fig. 2:**
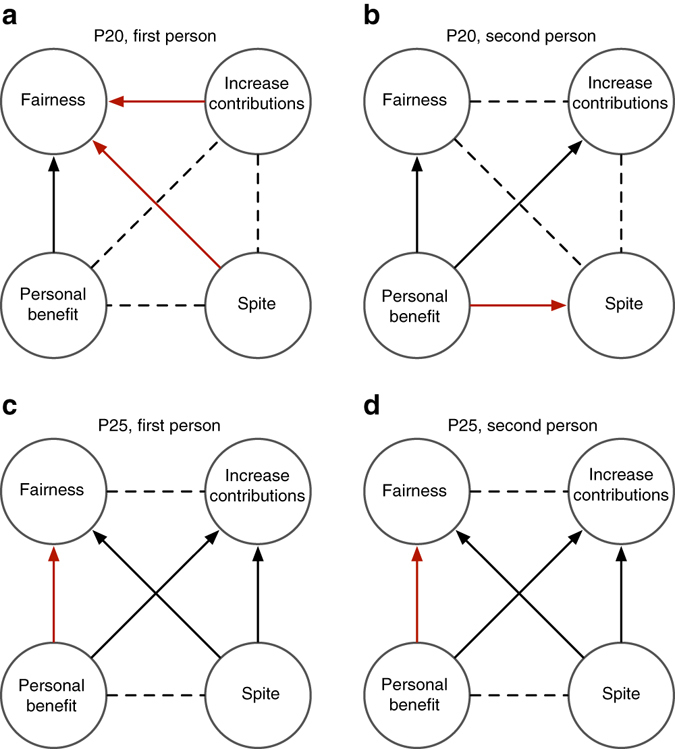
Relative frequency of reasons cited to explain punishment. First person reasons are responses to: ‘What was the main reason that you deducted points from the other members in your group?’ Second person reasons are responses to: ‘What do you think is the main reason that others may have deducted points from you?’ *Solid arrows* point to reasons cited significantly more frequently than the alternative, at the group level (two-tailed binomial test; *red arrows p* < 0.1, *black arrows p* < 0.05; *dashed lines* NS). The more prosocially oriented reasons, fairness and to increase contributions, are in the *upper circles* of each figure. P20 (**a**, **b**), *n* = 15; P25 (**c**, **d**), *n* = 14

One possible explanation for punishment behaviour—that subjects engaged in retaliatory counter-punishment for having been punished in earlier rounds—was precluded by the design of our experiment. Subjects were not advised who had punished them on any given round and subject identifiers were randomly assigned every round, making it difficult to identify who was responsible for past punishment received. A post-experiment survey asked subjects to explain why they penalised other players (first person punishment) and to indicate why they believed others may have penalised them (second person punishment). In both punishment treatments, there was negligible evidence of retaliatory motives and the reasons most commonly cited were reasons relating to fairness and increasing contributions, as opposed to personal benefit or spite, consistent with our hypothesis that normative motivations were a significant factor (see Fig. 2, also Supplementary Table 2 and Supplementary Fig. 2).

The evidence from our post-experimental survey, however, is open to alternative interpretations about normative motivations. Punishment may have given rise to higher levels of contributions if some subjects used the threat of penalties to extort contributions from other group members. This behaviour would still be consistent with an attempt to ‘increase contributions’, but would not be a normative motivation, because it makes no reference to the beliefs of others as to what is socially or morally appropriate. One might further doubt the veracity of any explanations offered after the experiment, given that they may reflect self-serving biases.

Consequently, to determine whether the subjects in our original experiment shared a relevant norm, we conducted a second experiment, in which subjects were shown the instructions from either the P20 or N20 treatments of the first experiment and then asked to identify the appropriateness of each possible contribution level. We restricted our second experiment to an investigation of the MPCR = 0.2 environment, because this is the setting in which the existence of a norm requiring positive contributions is outright destructive and hence most perverse.

Social norms can influence behaviour, even among those who reject the norm, because many agents regard it as important to be seen to do the right thing by the lights of the broader community^2, 3, 24, 25^. In eliciting beliefs about norms, it is therefore important to find out what each subject thinks the other subjects believe, rather than to elicit individual opinions about what is right. Adapting a methodology that has been successfully used to study second-order beliefs about norms in other settings^26, 27^, we gave subjects an incentive to accurately identify what they believed other subjects believed by offering a monetary reward for identifying the response which was given most frequently.

To determine whether normative beliefs are liable to vary across the treatment settings or to change through the course of play, we employed four treatments in a two-by-two design. In the ‘zero-base’ treatments, P20z and N20z, subjects read the instructions from the original experiment and were asked to evaluate the appropriateness of each possible contribution in the very first round. In the ‘history’ treatments, P20h and N20h, subjects read the same instructions but were also shown a sequence of play that actually occurred in the first 19 rounds of the initial experiment. Subjects were then asked to evaluate the appropriateness of all possible contributions in the final round.

**Fig. 3:**
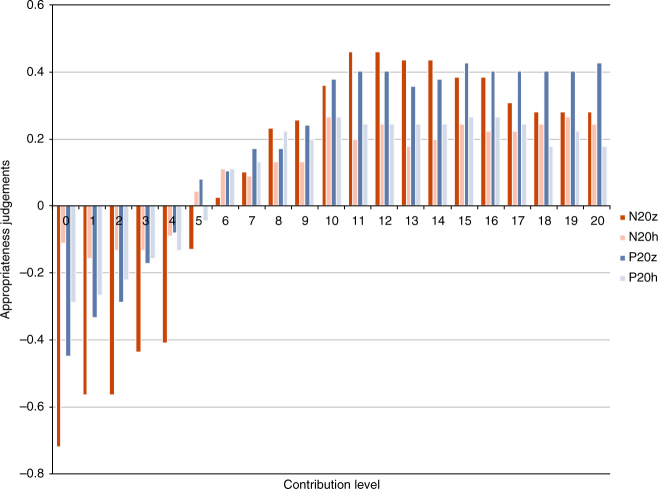
Mean appropriateness scores for all contribution levels, for each treatment. In all treatments, subjects tend to judge contributions levels > 5 as appropriate and < 5 as inappropriate. Numerical scores are derived from a linear scaling of subject’s categorical responses on a Likert scale ranging from −1 (very inappropriate) to +1 (very appropriate). N for the treatments: N20z = 26, N20h = 30, P20z = 29, P20h = 30

**Fig. 4:**
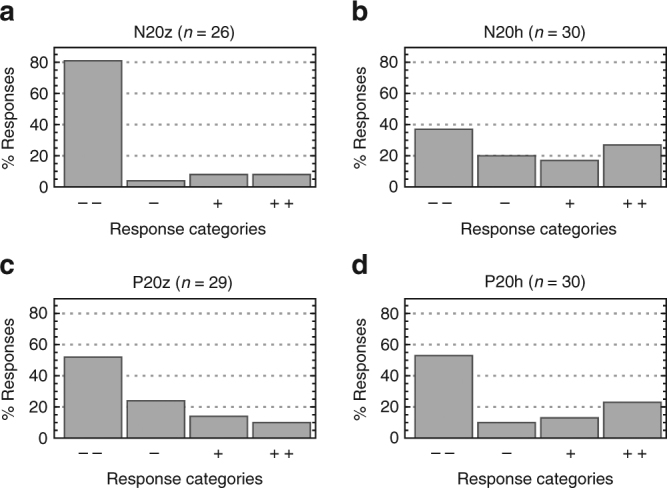
Distribution of normative attitudes towards contributing zero. Responses are ‘very socially inappropriate’ (––); ‘somewhat socially inappropriate’ (–); ‘somewhat socially appropriate’ (+); ‘very socially appropriate’ (++). In all treatments, more than half of respondents rate contributing zero as at least somewhat inappropriate. Treatments: **a** N20z; **b** N20h; **c** P20z; **d** P20h

We find compelling evidence that there was a norm requiring positive contributions. Subjects ranked the appropriateness of each possible contribution on a four-point Likert scale, which we converted to numerical scores on a linear scale from − 1 (very inappropriate) to + 1 (very appropriate). Average appropriateness scores for all contributions are displayed in Fig. 3. There is a consistent pattern across all treatments: low contributions are regarded as inappropriate, whereas high contributions are regarded as appropriate. Aggregating all our treatments, we observe that more than half of subjects rated contributing zero as very inappropriate. Half of subjects rated contributing one, two or three units as at least somewhat inappropriate and one quarter rated these as very inappropriate. Conversely, half of subjects rated contributions at every level greater than nine as at least somewhat appropriate and at least one quarter of subjects rated these positive contribution levels as very appropriate. The average appropriateness assessment given across all positive contribution levels is significantly higher than the appropriateness of making a zero contribution (Wilcoxon signed-rank test, *z* = –4.554, *p* < 0.0001). Conducting the same test within treatments, we found that in the zero base treatments, the same difference in attitudes was statistically significant (P20z: *z* = –3.181, *p* = 0.002; N20z: *z* = –3.684, *p* = 0.0002), but that it did not achieve significance within the history treatments (see also Supplementary Table 3). We further sought to investigate whether normative attitudes were affected by either the opportunity to punish or the observation of earlier rounds of play. To do this, we compared the distribution of normative attitudes towards contributing zero in each treatment (see Fig. 4). Contributing zero is a salient option to analyse, because it is not only uniquely efficient but also the contribution level that was most frequently punished, thus it is a focal point for the tension between efficiency and normative expectations. Subjects are more approving of contributing zero in the history treatments compared to the respective zero base treatments, though this is only statistically significant for N20h vs. N20z (Mann–Whitney test, *z* = –3.105, *p* < 0.002). This can presumably be ascribed to an intrinsic difference in normative attitudes towards contributions at the beginning vs. the end of the experiment, or to a learning effect experienced by subjects who are exposed to histories of play: subjects may be coming to disapprove of the inefficiency of the norm or they may be adjusting their belief about what others expect in light of the decline in contributions that happens over time. A similar pattern is observed for most contribution levels: normative attitudes (both positive and negative) are less extreme in the history treatments. The decline is only significant, however, for contribution levels 1 through 4 and only in non-punishment treatments. This is suggestive that punishment is stabilising the normative expectations against a tendency to otherwise decay.

**Fig. 5:**
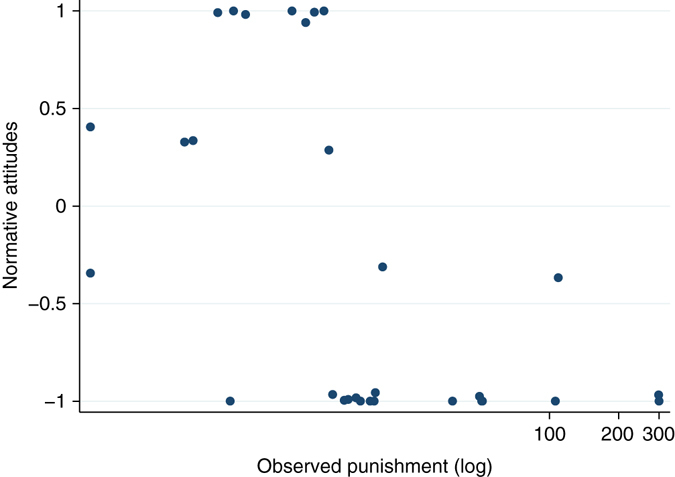
Normative attitudes towards contributing zero and total punishment observed in the P20h treatment. Points have been jittered to distinguish overlapping observations. *n* = 30, Spearman’s *ρ* = –0.665

There is further evidence that punishment is stabilising normative attitudes, derived from a closer examination of the P20h treatment. Subjects in history treatments were shown courses of interaction that actually occurred in the first 19 rounds of Experiment 1 (each of the 15 group histories was shown to precisely 2 subjects in Experiment 2), so subjects in P20h saw a variety of different amounts of punishment dispensed (mean 39, median 12, ranging from 1 to 277). Figure 5 shows the association between the total amount of punishment observed by subjects and their normative attitude towards contributing zero. A linear regression on this data is of doubtful value, given the small number of observations, and the highly skewed distribution of punishment levels, but a non-parametric test of association such as Spearman’s rank correlation confirms that the data are almost certainly associated: subjects who observe higher punishment levels tend to have stronger judgments condemning zero contributions as inappropriate (*ρ* = –0.665, *p* = 0.0001, *n* = 30).

## Discussion

Our key finding is that in the presence of punishment opportunities, destructive and inefficient norms can exert a significant influence on behaviour. This is important and surprising, not least because the experiment involves only a very subtle modification of a design that powerfully demonstrates the cooperative benefits of punishment^9^. Our results show that punishment supports not merely cooperative behaviour, but also destructive behaviour. These results are consistent with theoretical findings that punishment can stabilise any social equilibrium, cooperative or destructive^15, 28^ and previous experimental work on destructive behaviour^19^.

Three further questions arise: first, are there alternative explanations of our observations, not involving social norms? Second, what was the content of the destructive norm that emerged? Third, how robust is the long-term stability of destructive norms?

We have observed not only that subjects sometimes contributed, but that those who contributed tended to punish non-contributors also. We further observed a relatively non-normal distribution of contribution levels. Many individuals contributed very low amounts, some contributed very high amounts, but relatively few contributed intermediate amounts (see Supplementary Fig. 3).

This behaviour could be the result of a certain sort of social preference, without necessarily involving a social norm. The hallmarks of a social norm are that (i) a sufficiently large proportion of the population approves of a certain sort of behaviour, conditional on others having similar attitudes, and (ii) a sufficiently large proportion of the population (not necessarily the same proportion as in (i)) disapproves of non-compliance, and may be willing to sanction non-compliance^2^. Social norms are therefore highly suited to explaining behavioural regularities that are enforced by punishment.

An alternative possibility is that the population has a widely shared, but ‘unconditional’ attitude. For instance, many subjects may approve of generous behaviour, both by themselves and their fellow group members, but held this attitude regardless of their fellow group members’ attitudes. Punishment may be used by such subjects both to express their approval and to change the behaviour of fellow group members. This second possibility is what we might call a ‘moral’ commitment: moral ideals are–at least in theory^29, 30^–supposed to be especially robust, even in the face of community disagreement. Thus a moral revolutionary can have moral ideals that are at odds with the broader community, whereas it is not conceptually possible to have social norms that are at odds with the entire broader community (although of course it is possible to have subcultures that have distinctive norms—the point is that there must be some community whose consensus constitutes the norm).

The technique we used to elicit normative attitudes does not reliably discriminate between these possibilities. Therefore, we cannot rule out the possibility of a moral commitment explaining the behaviour we observe. However, one signature of a social norm being present is that it will tend to turn a social dilemma into something like a coordination game^2, 24, 31, 32^. Because of the interdependent structure of preferences, there will be multiple equilibria: typically a high-compliance equilibrium and a low-compliance equilibrium. At the high-compliance equilibrium, subjects believe that other group members will comply, and so to avoid the stigma of non-compliance, it is a best response to comply. At the low-compliance equilibrium, subjects believe that very few other group members will comply, and while there may be some honour involved in compliance, there is no shame in not complying. Thus, for most subjects, the best response is to not comply. Subjects who are moved by a moral commitment, however, may continue to contribute, even when no one else in the group is doing so. Given some heterogeneity in moral commitments, we would therefore expect to see behaviour that does not resemble a coordination equilibrium, but rather contribution behaviour distributed randomly across groups. The most extreme sort of moral commitment would be completely insensitive, not only to the contributions of other players, but also to the punishment decisions. This would predict that the distribution of contributions should be randomly distributed across groups.

This toy prediction, however, is not only unrealistic in its assumption that players are insensitive to punishment, it also neglects the possibility that a moral commitment may involve some concept of reciprocity. The reciprocator thinks that ‘one good turn deserves another’ and perhaps unkind behaviour deserves retaliation also^33, 34^, so a preference for reciprocity is a preference to behave kindly or harshly, conditional on the behaviour of others. The distinction between an unconditional preference for conditional generosity (i.e., a moral commitment to reciprocity) vs. a conditional preference for unconditional generosity (i.e., a social norm of generosity) is far more subtle than most experimental methods can detect. We do not insist that either is superior for the purposes of explaining our present findings (see the Supplementary Discussion for further remarks that relate our findings to extant models of social norms and social preferences). Rather, we suggest that both social norms and moral commitments are likely to be involved in the complete explanation of the observed phenomena. Morally committed individuals, who are willing to make contributions independently of community attitudes, are likely to play an important role in initiating an expectation that contributions are appropriate. The example they set is then liable to affect the conditional attitudes of those who lack moral commitments, but are more concerned to comply with whatever is approved of by the majority.

Regarding the content of the norm, as we have already alluded to above, the present study does not offer decisive evidence in this regard. That said, there are two salient possibilities that seem likely to have played a role in explaining the findings. The first is a norm related to fairness: the thought being something like, ‘If I am contributing, you should make a similar contribution, otherwise you’re benefiting at my expense’. This sort of norm is thought to be operative in typical cooperative settings^35–37^, and is naturally extended to the present context. Norms that draw on notions of fairness crucially involve a reciprocal element: what is required of one agent depends on what they have received from other agents. The second possibility dispenses with this conditional element: a norm of generosity simply requires that those who have resources share them with others. This sort of norm appears to be operative in asymmetric paradigms such as dictator games, and subjects have been observed making positive contributions even in inefficient dictator games, where each unit sacrificed by the dictator yields a diminished return for the recipient^38^. Both hypotheses are consistent with the survey responses we obtained, and discriminating further between these hypotheses may be difficult, because subjects themselves may not be certain precisely what norm is operating in this setting. Given the very limited social and contextual cues available to subjects in a laboratory setting, and given the limited means of communication, the evidence that subjects normally use to identify social norms is diminished.

It may be perplexing that either a norm of fairness or of generosity requires behaviour that does not benefit—and may even harm—the group as a whole, but it should not be surprising to think that norm-followers may be somewhat blind to the consequences of their behaviour. Social norms rely on general assent and informal, decentralised enforcement. In comparison with formal rules such as laws and regulations, there will be inherent limits on their complexity and on the ability of a population to change its norms to adapt to novel circumstances^39^. Norms therefore rely on familiar and intelligible structures, such as prohibitions on actions, rather than assessment of consequences^2, 40^. In the present case, contributing is indeed an example of generous behaviour: it benefits the other group members, although at some cost to the donor. (Indeed, it resembles institutions like the exchange of gifts at Christmas, which is similarly both generous and inefficient.) If some, but not all, group members make contributions, then it also fits a simple description of ‘unfairness’: some group members are benefiting at the expense of others. Therefore, it is not entirely surprising that at least some subjects think that contributing is required by one or both of these norms. Given the opportunity to express this attitude via punishment, these individuals may entrench the norm, to everyone’s detriment.

The apparently rigid and unthinking adherence to an inefficient norm is also illustrative of a more general phenomenon regarding norm enforcement. It is widely observed that punitive judgments are insensitive to consequences, and can be best explained by a retributive ‘eye for an eye’ type of rule that matches the degree of punishment with the degree of transgression^41, 42^. Game theoretic considerations suggest that retaliatory behaviour has to be inflexible in this manner or it will be ineffective^43, 44^. Similarly, in the present experiment, agents who enforce norms only when it is efficient to do so will be unable to use costly punishment at all (because punishment is inherently inefficient in the short term). This can be helpful where punishment is used to enforce norms that are socially beneficial, but can also lead to a disturbingly rigid commitment to whatever norms happen to arise. Our findings therefore suggest that once a norm is established through punishment, it can remain stable even though it may be collectively harmful. Repugnant customs may persist without observable coercion because of the mere existence of members of the group who are willing to punish non-compliers^2, 37^. Extending this research and understanding the stability of bad norms in the face of punishment will be crucial for understanding these social norms outside the lab and, most importantly, how they might be changed.

## Methods

### Experiment 1

The basis of our analysis is the long-run public goods game with and without punishment^1^. This game is typically used to test how the presence of punishment can change the incentives to free-ride or contribute to some shared public good. In the classic public goods game, the returns for each player are greater if each contributes to the group account, but there is also an incentive to not contribute and to still reap the benefits of the public good. Real world examples of this scenario include the use of open access lands for the grazing of livestock and environmental degradation, the management of shared water resources and irrigation, and more speculative examples such as the development of altruism and social institutions. The robust lab result of these experiments is that the threat of punishment, either from third-parties^37^ or from within the group^1, 9, 22^, will reliably encourage greater contribution to the public resource and stabilise cooperation over time. There are also, however, theoretical models yet to be experimentally tested^15^, which show that punishment can enforce any kind of group norm, whether cooperative or not. To test this idea, we created a non-efficient group contribution game to see if punishment would enforce bad, inefficient, norms, as well as cooperative ones.

Our design closely followed the paradigm of Gächter et al.^9^, with the exceptions that: (i) our groups consisted of four players rather than three and (ii) the rate of return is lowered, so depending on the treatment, contribution has either no effect on group payoff (treatment P25, MPCR = 0.25) or is inefficient (P20, MPCR = 0.2).

The game lasts for 20 rounds and at the beginning of each round players are endowed with 30 MU (AUD $1.20) in addition to a AUD $8 show up fee. In each round, players may contribute up to 20 MU to a group account with either a MPCR of 0.25 or 0.20. In the first treatment (MPCR 0.25), there is no group benefit to making contributions and in the second treatment (MPCR 0.20), the expected return is negative. (The additional 10 MU endowment that cannot be contributed to the group account is to ensure a reserve of funds which players in punishment treatments can spend on penalising other players.)

Investment decisions were made simultaneously and subjects were not able to communicate with one another. Subjects were informed after each round of the contributions of other group members. The identifiers for the other players in a group were randomly reassigned in each round, such that counter-punishment across rounds was ruled out.

After contributions were made and shown to the group, players were able to assign penalty points (referred to as deduction points in the instructions) to other players in their group. Penalty points cost 1 MU (AUD $0.04) to assign and reduce the earnings of the player they are assigned to by 3 MU (AUD $0.12). Both the no-benefit (MPCR 0.25) and negative-return (MPCR 0.20) treatments were run with a penalty round and a baseline treatment where players are not able to assign penalty points. There are four total treatments with 20 rounds each: no-benefit treatments with punishment (P25) and without (N25), as well as negative-return treatments with punishment (P20) and without (N20).

In Gächter et al.^9^, the participants are either in a 10-round or 50-round treatment and found that there was little change in the later rounds after 20 rounds. We therefore concluded that 10 rounds is too few observations, whereas 50 rounds seems to be unnecessary and settled on 20 rounds as an appropriate number. Our results suggest that even this may have been unnecessary as in both the punishment and no-punishment treatments in the negative-return cases group behaviour stabilises around round 10.

We collected data from a total of 232 subjects who participated in 58 groups. Subjects earned an average of AUD 35.68, including an AUD 8 show-up fee. All the interactions in the experiment took place anonymously via computer terminals. We used the experimental software z-Tree on computers in the Monash Laboratory for Experimental Economics (MonLEE) laboratory^45^. ORSEE software was used to recruit subjects^46^. The instructions provided to subjects are provided in Supplementary Note 1.

We conducted our experiment at the MonLEE at Monash University in Melbourne, Australia. The subject pool was relatively balanced between genders, with 112 females and 118 males. The average age of our subjects was 22.5 years. Subjects were all students, and described their areas of study as Engineering (30%), Economics (9%), Other Business (19%), Sciences (14%), Psychology (2%) and Other (26%).

After arrival, the participants were randomly assigned to computer terminals separated by barriers to preserve anonymity. Subjects were then presented with a set of written instructions, which were read aloud to the entire group. Subjects were then required to solve several quiz questions (Supplementary Note 3) on their terminals and the group session would not begin until every member of the group had successfully completed these questions. The quiz was conducted to make sure that all subjects understood the instructions and how their payoffs were calculated.

After the public goods game, we administered an anonymous survey to the subjects (final earnings included compensation for their time spent answering the questions). The survey helped us obtain general demographic information and responses to questions intended to elicit information about subjects’ normative attitudes towards levels of contribution. Two of the questions invited qualitative responses. To analyse this data, a research assistant who was blind to the research questions of this study was invited to code the answers on the basis of whether they cited reasons such as fairness, personal benefit, or revenge. The survey questions, the instructions for the research assistant and the qualitative responses to the survey questions are provided in Supplementary Notes 4–6.

The experiment took ~1 h for the punishment treatments and around half an hour for the non-punishment treatments.

### Experiment 2

One hundred and fifteen subjects played a pure matching coordination game^44^, in which they were rewarded for picking the most commonly chosen rating of evaluation for a possible action in one of the treatments of the first experiment. Subjects read the instructions of the N20 or P20 treatments from Experiment 1 and were then given a quiz to test comprehension of the game. Subjects in history treatments were asked to identify, on a four-point Likert scale, the appropriateness of all possible contribution levels on round 20, having been shown an actual history from the original experiment. Subjects in zero-base treatments made the corresponding assessment for all possible contributions in round 1. The instructions provided to subjects are given in Supplementary Note 2. In the history treatments, each group history from experiment 1 was shown to precisely two subjects, ensuring that responses reflected a representative exposure to the behaviour that occurred in experiment 1.

One of the response items was randomly chosen to be the basis for reward. Subjects earned $10 for participation and an additional $10 for choosing the most common response on this item.

The study design was approved by the Monash University Human Research Ethics Committee, approval no. CF15/1440–2015000693. Informed consent was obtained from all subjects.

### Statistical methods

The statistics presented in the main paper are all non-parametric tests and thus do not make any distributional assumptions about the data.

### Data availability

All data on which the findings of this study are based are available at https://figshare.com/s/1dc581ff601213c35e83.

## Electronic supplementary material

Supplementary Information
