# Supplementary material for: Peer punishment promotes enforcement of bad social norms
Source: Nat Commun. 2017 Sep 20;8:609. doi: 10.1038/s41467-017-00731-0 (PMC5607004; doi:10.1038/s41467-017-00731-0)
Supplement: Supplementary file 1 — Supplementary Information [file 41467_2017_731_MOESM1_ESM.pdf]

File name: Supplementary Information
Description: Supplementary Figures, Supplementary Tables, Supplementary Notes, Supplementary Discussion and Supplementary References

Supplementary Tables

	(1) Punishment received	(2) Punishment dispensed
Constant	3.580	3.784
	(3.701)	(3.805)
Others' average contributions	0.018	0.019
	(0.018)	(0.019)
Negative deviation (abs. value)	0.047**	0.142
	(0.166)	(0.144)
Positive deviation	0.048	0.281*
	(0.073)	(0.168)
Treatment dummy for MPCR = 0.2	2.1	2.046
	(5.326)	(5.359)
Number of observations	116	116
R-squared	0.254	0.163
F(4, 28); Prob > F	0.0001	0.346

Supplementary Table 1. Regression models of punishment received (column 1) and punishment dispensed (column 2). The independent variables include others' average contribution, the positive and negative deviations from others' average contributions, and the treatment dummy. We observe that negative deviation affects punishment received significantly (p = 0.02). In particular, the more an individual's contribution falls short of the average, the more he or she gets punished. Also, consistent with intuition and the previous literature, most punishment is meted out by high contributors (Column 2). In a regression model, in which the total punishment points given out is the dependent variable, we find that those who deviate positively from others' average contributions dispense more punishment points, though the effect is only marginally statistically significant (p = 0.10). Standard errors are given in parentheses. * denotes significance at the 10-percent level, ** at the 5-percent level. The regression models report coefficients from an ordinary least squares regression. These are for the punishment treatments only and are clustered at the group level. Regression results based on Tobit models are similar.

	First person punishment		Second person punishment	
Reasons	P20	P25	P20	P25
Fairness vs personal benefit	8:1**	9:2*	9:0**	9:2*
Fairness vs spite	9:2*	9:1**	7:4	8:1**
Fairness vs encourage contributions	10:3*	3:4	4:4	3:6
Encourage contributions vs personal benefit	5:4	12:1**	8:0**	12:1**
Encourage contributions vs spite	6:3	9:0**	7:4	9:0**
Spite vs personal benefit	4:5	4:4	10:3*	4:4

Supplementary Table 2. Comparative ratios of groups in which one reason was cited more often than the alternative, to explain first person or second person punishment. * denotes the null hypothesis that both reasons are equally likely to be cited is rejected at $p < 0.1$. ** denotes $p < 0.05$. Binomial test, two-tailed.

	p-values			
Contribute zero versus	N20z	N20h	P20z	P20h
Contribute five	<.001	.30	<.001	.11
Contribute ten	<.001	.15	<.01	.05
Contribute fifteen	<.001	.21	<.01	.07
Contribute twenty	<.001	.20	<.01	.11
n	26	30	29	30

Supplementary Table 3. Testing for within-subjects differences in normative attitudes towards contributing zero versus making positive contributions. For the zero-base treatments (N20z and P20z), subjects hold significantly different attitudes towards contributing zero versus positive contribution levels, viz. they judge contributing zero as significantly less appropriate. The differences in attitude are marginally significant for some contribution levels in P20h, and never significant for N20h. Similar results hold for other positive contribution levels greater than five. (Wilcoxon signed rank tests.)

Supplementary Figures

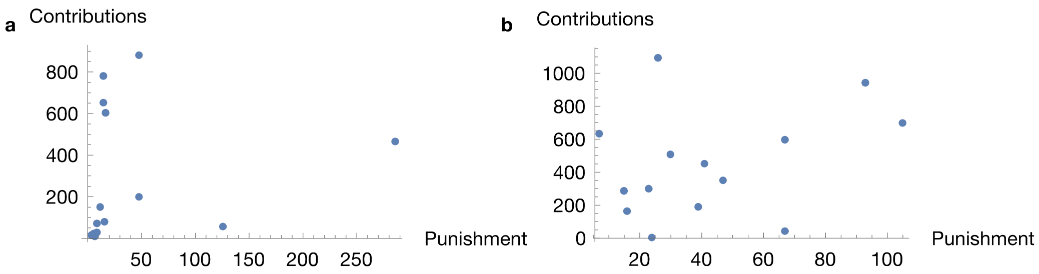

Supplementary Figure 1. Scatter plot of total contributions against total punishment dispensed at group level. **a** P20 treatment, Spearman ρ = 0.701 (n = 15, p = 0.004). **b** P25 treatment, Spearman ρ = 0.297 (n = 14, p = n/s). The Spearman's rank correlation coefficient (ρ) is a non-parametric measure of rank correlation which assesses the dependence of two vectors, based on ordinal rank information. This coefficient will be high when observations have a similar rank between two variables, and low when observations have a dissimilar rank between the two variables. In both treatments, the cofficient is positive, and in P20 the null hypothesis that the variables are independent is rejected with $p < 0.01$; the same test is not significant for P25. A linear regression on contributions against punishment did not yield significant results for either treatment. Although suggestive that punishment is important in maintaining high contributions, the data do not support a categorical finding.

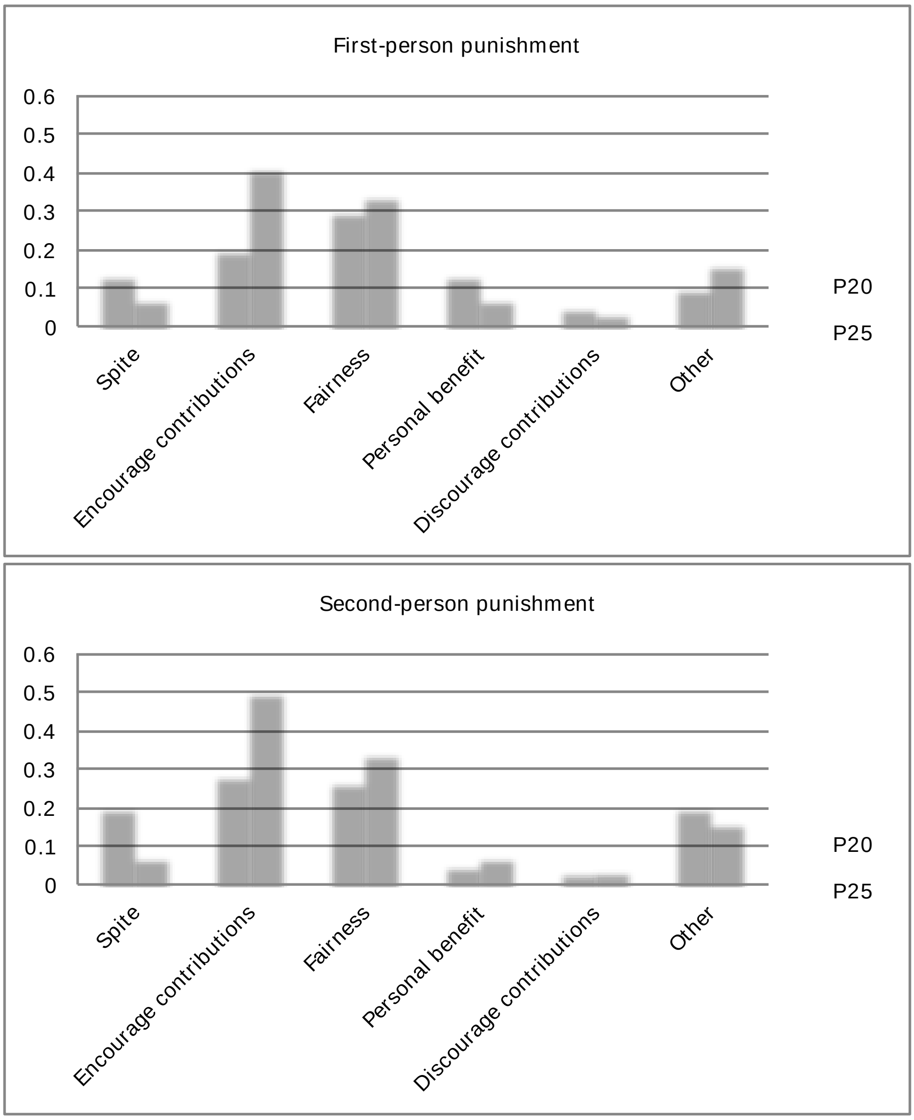

Supplementary Figure 2. Frequency of reasons cited by individuals for punishment dispensed by respondent (first-person punishment) and punishment dispensed by others to the respondent (second-person punishment). P20, n = 60; P25, n = 56. The category 'encourage contributions' merges categories originally coded separately as 'encourage high contributions' and 'discourage low contributions'. (See Supplementary Methods for original instructions given for coding of responses.)

a

b

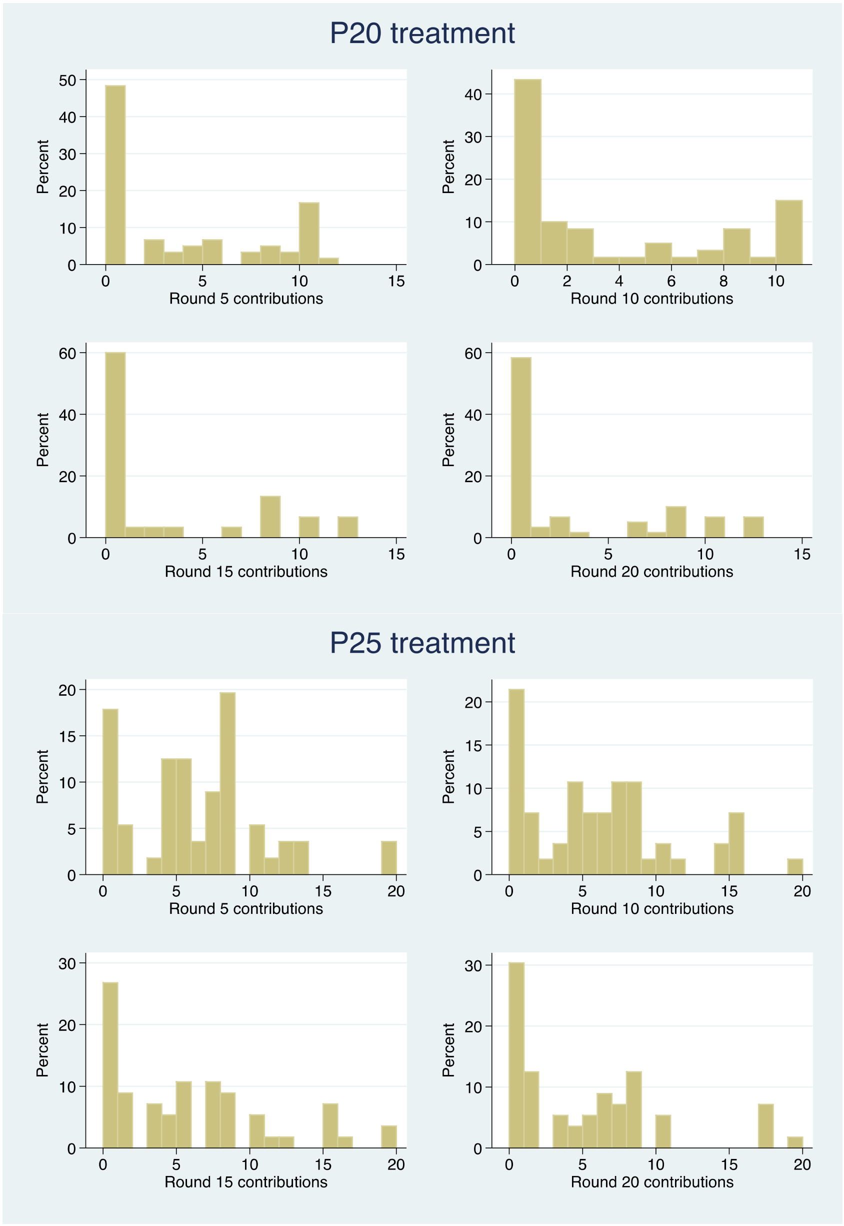

Supplementary Figure 3. Histograms of individual contributions at various stages in P20 treatment (**a**) and P25 treatment (**b**).

Supplementary discussion

In these remarks, we evaluate the potential of some representative models of social preferences (broadly construed) to predict our observations.

Rabin's model of reciprocity [1] invokes a level of behaviour that is regarded as the default, and behaviour that deviates from this baseline is regarded as 'kind' or 'unkind', depending on its effect on others. Adapting a refinement of Rabin's model that is more appropriate for a four-player setting [2], the utility of player i from contributing x_i, given the other players give $\mathbf{x}_{j \neq i}$, is

$$U(x_i | \mathbf{x}_{j \neq i}) = (30 - x_i) + 0.2\left(x_i + \sum_{j \neq i} x_j\right) + Y_i\left(0.05\left(\sum_{j \neq i}(x_j - a)\right)\right)(1 + 0.05(x_i - a))$$

where a is the neutral action, i.e. contributions above a are considered kind and contributions below a are considered unkind. The first term reflects the endowment player i retains after any contributions. The second terms reflects earnings from the group account. We consider here the case of MPCR = 0.2. The third term reflects the payoff from reciprocity, weighted by parameter Y_i. (The coefficient 0.05 reflects the MPCR divided by the number of players.) The third term will be positive when player i reciprocates the unkindness of others (the other players contribute less than a on average, and so too does i) but also when player i reciprocates the kindness of others (the others contribute more than a on average, and so too does i.

This model can be read as a description of a 'moral commitment', given that the utility function makes no reference to the preferences of others. However, to the extent that the neutral behaviour a may itself be a social convention, the model may also be regarded as assuming a social norm is already in operation.

The first-order derivative of U is

$$\frac{\partial U}{\partial x_i} = -0.8 + 0.0025Y_i\left(\sum_{j\neq i}(x_j - a)\right)$$

Note that, strictly speaking, because the payoff function is linear in x_i, the model generally predicts players contribute either everything or nothing, depending on whether the above expression is positive or negative. This depends on variables that are outside player i's control; the strategic variable x_i does not enter the first order condition.

An interior equilibrium could only emerge where the above derivative is zero. If we consider what symmetric behaviour (all players contribute the same amount x) would be an equilibrium, the necessary condition for a reciprocity equilibrium is

$$Y_i = \frac{106.67}{x - a}$$

Thus, to sustain a reciprocity equilibrium in which everybody contributes x, given the neutral action is a, the weighting of the reciprocity component must be $106.67/(x - a)$. In the parameterisation of the experiment, reciprocity considerations alone could only explain our data if players receive a utility from reciprocity that is very high. If we make the most favourable assumption, that a = 0, then it still requires Y_i in the order of 10, which is an order of magnitude greater than what has been found when this model has been applied elsewhere.

Even at this equilibrium, because of the linearity of the model, the equilibrium would be utterly unstable. So while solving for x can yield a symmetric equilibrium in which every player's action is a best response to every other player's choice, deviating from the equilibrium choice bears no loss of utility, such that the emergence of this equilibrium would be entirely coincidental. The model thus has little interest as a predictive device in this setting.

Turning to a model that looks initially more promising to capture the notion of a social norm, Krupka and Weber [3] define an appropriateness function N, which captures how socially appropriate a choice is. Appropriateness then enters a player's utility function via a weighting parameter γ_i.

Our elicitation of appropriateness, as depicted in Figure 3, strongly suggests that the appropriateness function is piecewise linear, with a value of approximately −0.4 for contributions of 0, a linear rise to 0.4 for contributions up to 10, and a constant above that.

$$N(x_i) = \begin{cases} -0.4 + 0.08x_i, & x_i < 10 \\ 0.4, & x_i \geq 10 \end{cases}$$

A player's utility function can then be formulated as

$$U(x_i|\mathbf{x}_{j\neq i}) = (30 - x_i) + 0.2\left(x_i + \sum_{j\neq i} x_j\right) + \gamma_i N(x_i)$$

The first two terms are identical to those in Rabin's model, while the third term reflects the player's concern to make a socially appropriate contribution. After some rearrangements, we obtain

$$U(x_i|\boldsymbol{x}_{j\neq i}) = \begin{cases} 30 - (0.8 - 0.08\gamma_i)x_i + 0.2\sum_{j\neq i} x_j - 0.4\gamma_i, & x_i < 10 \\ 0 - 0.8x_i + 0.2\sum_{j\neq i} x_j - 0.4\gamma_i, & x_i \geq 10 \end{cases}$$

It can be seen immediately that there can be no interior equilibrium less than 10. For $x_i < 10$, utility either always decreases ($\gamma_i < 10$) or increases ($\gamma_i \geq 10$) in x_i. It cannot make sense to contribute more than 10, as increasing contributions beyond 10 decreases material payoff without gaining appropriateness. So the model predicts players will contribute either zero or 10. This is at least a better fit with our observations, whereby zero and 10 do appear to be focal points for contribution.

The absence of an interior equilibrium stems from two factors. First, the linear payoff structure of the standard public good paradigm we use, and second, the piecewise linear relationship between contributions and appropriateness that we find in our data. The artefact of linearity could of course be removed from both these models, but a more fundamental issue is that neither model directly represents the *conditional* structure of a social norm. If we combine either model with heterogeneity in sensitivity to normative considerations (i.e. in parameters Y and gamma, respectively), the models predict that some players will make maximal contributions, and others will make minimal ones, but we do not derive the prediction that high contributions will beget other high contributions – rather we would expect individual contributions to be randomly distributed across groups.

López-Pérez has provided a prominent example of a model of norm compliance[4] that begins to address this issue: agents' utility is dependent on whether or not they comply with the norm. For those that comply, they experience negative utility as a result of anger towards non-compliers, where this quantity is increasing with the payoff of the best remunerated cheat; those that do not comply experience negative utility as a result of shame, where this quantity increases with the number of other players that do comply. This sort of dependence on the number of others who comply is the best attempt of which we are aware to explicitly model the essential feature of a social norm. His model, however, simply captures a binary compliance/non-compliance distinction, rather than degrees of appropriateness, as we see in the Krupka–Weber model. Developing a tractable hybrid of these modelling approaches, and attempting to obtain testable predictions from the result, will be a fruitful area for future research, we suspect.

Supplementary Note 1: Experiment 1 instructions

Below are the instructions for the Baseline (no-punishment) negative-return treatment (N20) and the instructions for the negative return treatment with punishment (P20). The other two treatments (N25 and P25) differed only in the payoff amounts.

Instructions for Experiment 1 (N20, Negative-Return Baseline)

This is an experiment on decision-making. If you read the following instructions carefully, you can, depending on your decisions, earn a considerable amount of money. It is therefore very important that you read these instructions carefully.

Please do not communicate with the other participants during the experiment.

If you have a question at any time raise your hand and the monitor will come to your desk to answer it.

During the experiment you can earn "points" (or lose points). At the end of the experiment these points will be converted to cash at the following rate:

1 point = $0.04

Each participant receives a lump sum payment of **200 points** at the beginning of the experiment. This one-off payment can be used to pay for eventual losses during the experiment**. However, you can always avoid losses with certainty through your own decisions**. At the end of the experiment your entire earnings from the experiment plus the lump sum payment will be paid to you **in cash**.

The experiment is divided into different periods. In all, the experiment consists of **twenty** periods. In each period the participants are divided into groups of four. You will therefore be in a group with three other participants. The composition of the groups will stay the same for all twenty periods. **You are therefore with the same people in a group for all twenty periods.**

Each period consists of **a single stage**. In this stage you will be endowed with tokens (each worth one point) and have to decide how many tokens you would like to contribute to a project. The following pages describe the course of the experiment in detail:

Detailed Information on the Experiment

At the beginning of each period each participant receives an endowment of **30 tokens**. Your task is to decide how many of the 30 tokens you want to contribute to a **project** and how many of them to keep for yourself. The maximum number of tokens you can contribute is 20. The consequences of your decision are explained in detail below.

At the beginning of each period the following input-screen for the first stage will appear:

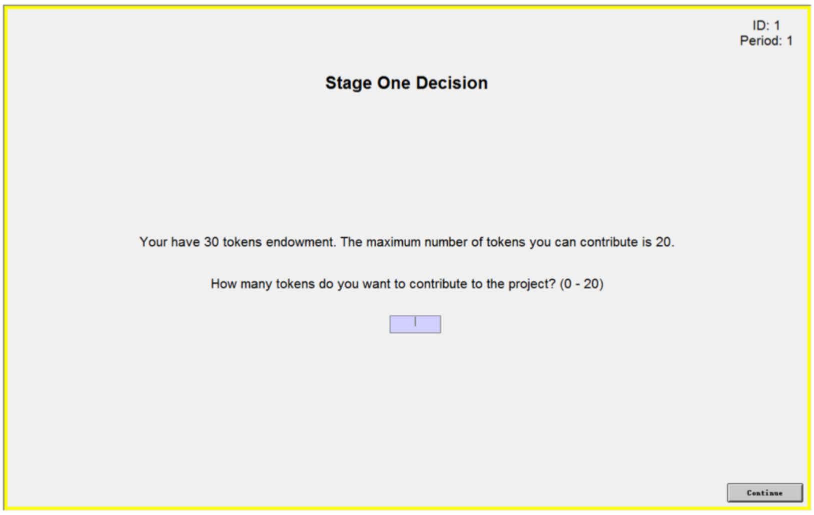

The First Stage Input Screen

Your **endowment in each period is 30 tokens**. You have to decide how many tokens (up to a maximum of 20) you want to contribute to the project by typing a number between 0 and 20 in the input field. This field can be reached by clicking it with the mouse. As soon as you have decided how many tokens to contribute to the project, you have also decided how many tokens you keep for yourself: This is (**30 – your contribution**) tokens. After entering your contribution you must press the CONTINUE button. Once you have done this, your decision has been made and cannot be changed.

After all members of your group have made their decisions the following income screen will show you how many points you have earned in the first stage.

The First Stage Income Screen

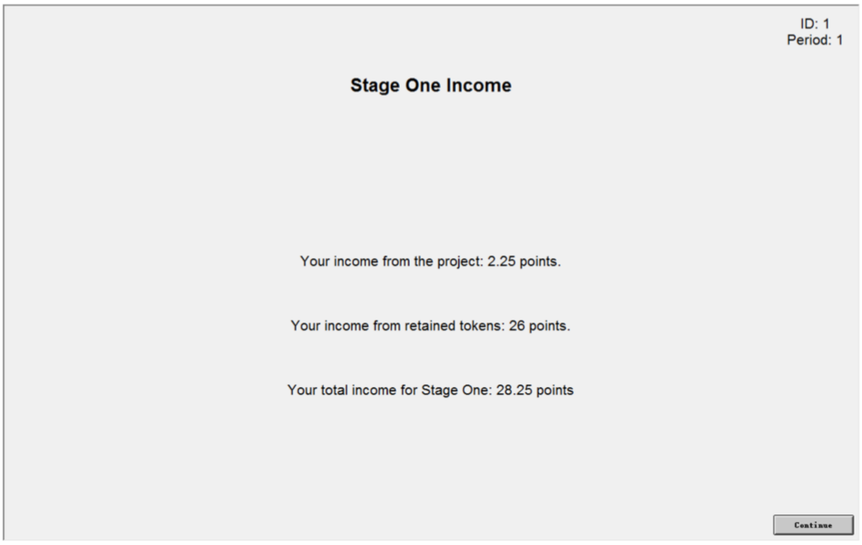

As you can see, your **income** consists of two parts:

(1) The tokens which you have kept for yourself ("Income from retained tokens") whereby **1 token = 1 point**.

(2) The "income from the project".

This income is calculated as follows:

Your income from the project = 0.20 *times* the total contributions to the project.

Your **income in points from the first stage** of a period is therefore:

(30 – your contribution to the project) + 0.20 × (total contributions to the project)

The income of each group member from the project is calculated in the same way, i.e., each group member receives the same income from the project. Assume, for example, that the sum of the contributions of all group members is 60 tokens. In this case each member of the group receives an income from the project of: 0.20 × 60 = 12 points. If

the total contribution to the project is 15 tokens, then you and all other group members receive an income of 0.20 × 15 = 3 points from the project.

For each token you keep for yourself you earn an income of 1 point. Supposing you contributed this token to the project instead, then the total contribution to the project would rise by one token. Your income from the project would rise by 0.20 × 1 = 0.20 points. However the income of the other group members would also rise by 0.20 points each, so that the total income of the group from the project would rise by 0.8 points. Your contribution to the project therefore also raises the income of the other group members.

Similarly, you earn income for each token contributed by the other members to the project. For each token contributed by any member you earn 0.20 × 1 = 0.20 points. After you have viewed the income screen the period is over and the next period commences.

Instructions for the experiment (P20, Negative Return with Punishment)

This is an experiment on decision-making. If you read the following instructions carefully, you can, depending on your decisions, earn a considerable amount of money. It is therefore very important that you read these instructions carefully.

Please do not communicate with the other participants during the experiment.

If you have a question at any time raise your hand and the monitor will come to your desk to answer it.

During the experiment you can earn "points" (or lose points). At the end of the experiment these points will be converted to cash at the following rate:

1 point = $0.04

Each participant receives a lump sum payment of **200 points** at the beginning of the experiment. This one-off payment can be used to pay for eventual losses during the experiment**. However, you can always avoid losses with certainty through your own decisions**. At the end of the experiment your entire earnings from the experiment plus the lump sum payment will be paid to you **in cash**.

The experiment is divided into different periods. In all, the experiment consists of **twenty** periods. In each period the participants are divided into groups of four. You will therefore be in a group with three other participants. The composition of the groups will stay the same for all twenty periods. **You are therefore with the same people in a group for all twenty periods.**

Each period consists of **two stages**. In the first stage you will be endowed with tokens (each worth one point) and have to decide how many tokens you would like to contribute to a project. In the second stage you will be informed of the contributions to the project by the three other group members. You will then decide whether or how much to reduce their earnings from the first stage by distributing **deduction points**. The following pages describe the course of the experiment in detail:

Detailed Information on the Experiment

The First Stage At the beginning of each period each participant receives an endowment of 30 tokens. Your task is to decide how many of the 30 tokens you want to contribute to a project and how many of them to keep for yourself. The maximum number of tokens you can contribute is 20. The consequences of your decision are explained in detail below.

At the beginning of each period the following input-screen for the first stage will appear:

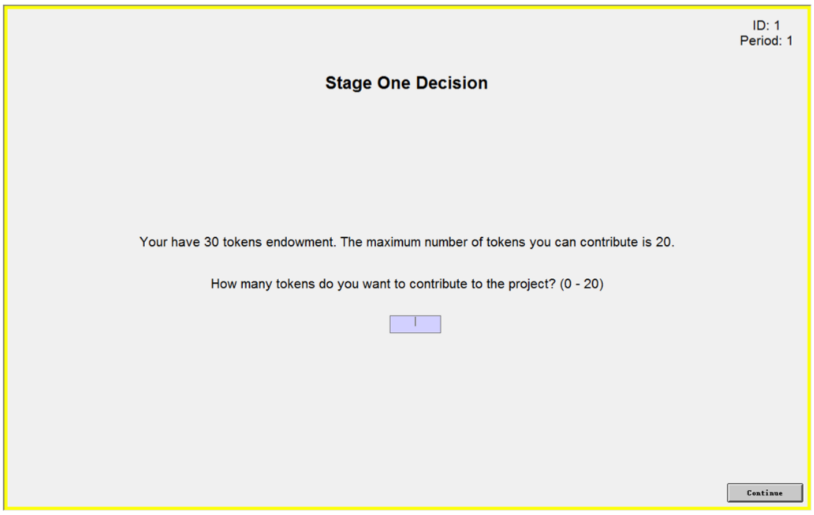

The First Stage Input Screen

Your endowment in each period is 30 tokens. You have to decide how many tokens (up to a maximum of 20) you want to contribute to the project by typing a number between 0 and 20 in the input field. This field can be reached by clicking it with the mouse. As soon as you have decided how many tokens to contribute to the project, you have also decided how many tokens you keep for yourself: This is (30 – your contribution) tokens. After entering your contribution you must press the CONTINUE button. Once you have done this, your decision has been made and cannot be changed.

After all members of your group have made their decisions the following income screen will show you how many points you have earned in the first stage.

The First Stage Income Screen

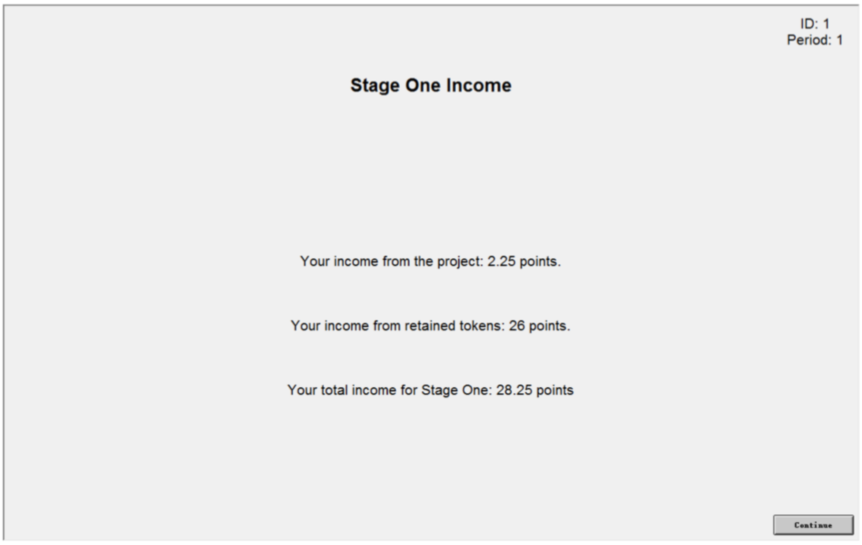

As you can see, your income consists of two parts:

(1) The tokens which you have kept for yourself ("Income from retained tokens") whereby 1 token = 1 point.

(2) The "income from the project". This income is calculated as follows:

Your income from the project = 0.20 *times* the total contributions to the project.

Your income in points from the first stage of a period is therefore:

(30 – your contribution to the project) + 0.20 × (total contributions to the project)

The income of each group member from the project is calculated in the same way, i.e., each group member receives the same income from the project. Assume, for example, that the sum of the contributions of all group members is 60 tokens. In this case each member of the group receives an income from the project of: 0.20 × 60 = 12 points. If the total contribution to the project is 15 tokens, then you and all other group members receive an income of 0.20 × 15 = 3 points from the project.

For each token you keep for yourself you earn an income of 1 point. Supposing you contributed this token to the project instead, then the total contribution to the project would rise by one token. Your income from the project would rise by 0.20 × 1 = 0.20 points. However the income of the other group members would also rise by 0.20 points each, so that the total income of the group from the project would rise by 0.8 points. Your contribution to the project therefore also raises the income of the other group members.

Similarly, you earn income for each token contributed by the other members to the project. For each token contributed by any member you earn 0.20 × 1 = 0.20 points. After you have viewed the income screen the first stage is over and the second stage commences.

The Second Stage

In the second stage you will see how much each group member contributed to the project. Moreover, in this stage you can decide whether to decrease the income of each other group member by assigning deduction points. The other group members can also decrease your income if they wish to. This is apparent from the input screen at the second stage:

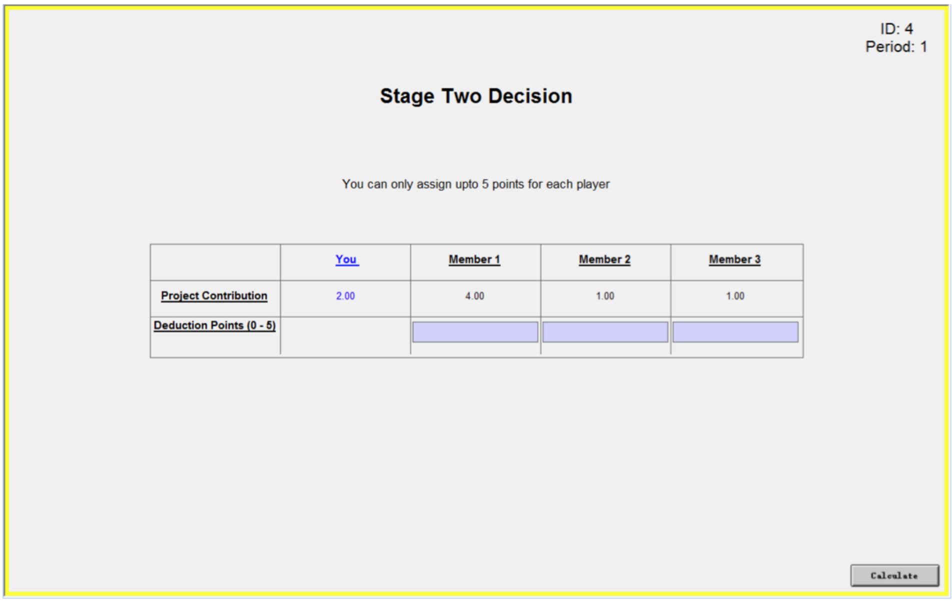

The Second Stage Input Screen

Your contribution is displayed in blue in the first column, while the contributions of the other group members of this period are shown in the remaining three columns. Note that the order in which others' contributions are displayed will be determined at random in every period. The contribution in the second column, for example, could represent a

different group member in different periods. The same holds true for the third and fourth column.

You will have to decide how many deduction points to assign to each of the other three group members. You must enter a number for each of them. If you do not wish to change the income of a specific group member then you must enter 0. You can assign up to 5 points to each group member.

You will incur costs from assigning deduction points. Every deduction point you assign costs you 1 point. For example, if you assign 2 deduction points to one member, this costs you 2 points; if, in addition, you assign 4 deduction points to the other member this costs you an additional 4 points. In total you will have assigned 6 points and your total costs therefore amount to 6 points.

After you have assigned points to each of the other three group members you must click the button "calculation" (see the second stage input screen). On the screen you will then see the total costs of your assigned points. As long as you have not yet clicked the OK button, you can still change your decision. To recalculate the costs after a change of your assigned points, simply press the "calculation" button again.

If you assign 0 deduction points to a particular group member (i.e., enter "0"), you will not alter his or her income. If you assign one deduction point to a group member you will decrease the income of this group member by 3 points. If you assign a group member 2 deduction points you will decrease the group member's income by 6 points, and so on. Each deduction point that you assign to another group member will reduce

his or her income by 3 points. Similarly, each deduction point assigned to you by another group member will reduce your first stage income by three points:

Costs of received deduction points = 3 × Sum of received deduction points.

How much the income at the second stage is decreased depends on the sum of deduction points received. For instance, if somebody receives a total of 3 deduction points (from all other group members in this period), his or her income would be decreased by 9 points. If somebody receives a total of 4 deduction points, his or her income is reduced by 12 points. It is possible that your income can be reduced below zero. If this happens your losses will be deducted from your initial lump sum payment.

Your total income from the two stages is therefore calculated as follows:

Total income (in points) at the end of the second stage = period income =

Income from first stage – 3 × (sum of received deduction points) – sum of deduction points you have assigned

After all participants have made their decision, your income from the period will be displayed on the following screen:

Income screen at the end of the second stage

After you have viewed the income screen the period is over and the next period commences.

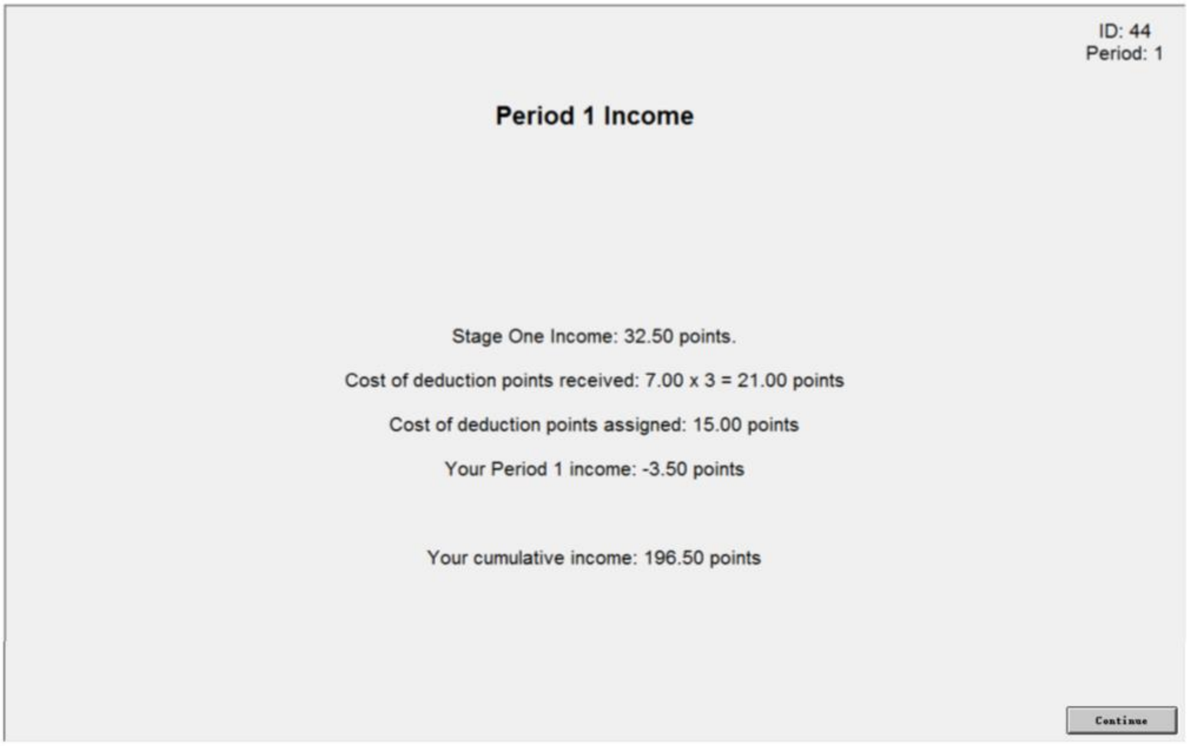
ID: 44
Period: 1
Period 1 Income
Stage One Income: 32.50 points.
Cost of deduction points received: 7.00 x 3 = 21.00 points
Cost of deduction points assigned: 15.00 points
Your Period 1 income: -3.50 points
Your cumulative income: 196.50 points
Continue

Supplementary Note 2: Experiment 2 instructions

Initial instructions, all treatments

On the following pages, you will read instructions from a real experiment that has been performed earlier. You will then read a description of the circumstances in which one of the experimental subjects, ``Individual A'', was required to make a decision. This description will include all possible choices available to Individual A.

After you read the description of the decision, you will be asked to evaluate the different possible choices available to Individual A and to decide, for each of the possible actions, whether taking that action would be ``socially appropriate'' and ``consistent with moral or proper social behavior'' or ``socially inappropriate'' and ``inconsistent with moral or proper social behavior.'' By socially appropriate, we mean behavior that most people agree is the ``correct'' or ``ethical'' thing to do. Another way to think about what we mean is that if Individual A were to select a socially inappropriate choice, then it might be reasonable for someone else to be angry at Individual A for doing so.

In each of your responses, we would like you to answer as truthfully as possible, based on your opinions of what you and all other experimental participants regard as socially appropriate or socially inappropriate behavior.

You have already earned $10 for your participation today. Based upon your decisions in the experiment, it is possible to increase your earnings, so please pay close attention to the instructions.

Survey instructions

On the following pages, we describe a situation that actually arose in the earlier experiment. We will ask you to consider the contribution decisions that one participant, Individual A, had to make in that situation.

You will receive a sheet, with a table on which to indicate your responses. The experimenter will read a description of the situation. You will then indicate whether each possible choice available to Individual A is socially appropriate or socially inappropriate.

At the end of the experiment today, we will select one of the questions you all answered, by randomly drawing a number from 1 to 21. For the question selected, we will determine which response was selected by the most people here today. **If you give the same response as that most frequently given by other people, then you will receive an additional $10.** This amount will be paid to you, in cash, at the conclusion of the experiment.

For instance, if we were to select the decision ``Contribute 5 tokens'' and most people in today's session indicated that this was ``somewhat socially appropriate'', then if you were one of the people who gave this answer to this question, you would earn an additional $10 (for a total payment today of $20). If you did not select the most common answer, you would receive only the $10 participation fee, and no additional payment.

If you have any questions from this point on, please raise your hand and wait for the experimenter to come to you. Please do not talk to the other experimental subjects.

Please turn the page to begin once the experimenter asks you to do so.

Zero-base instruction

The situation you are to consider is the beginning of the experiment, before any contributions have been made. Individual A has to make a decision how many tokens to contribute on the very first round.

The table below presents a list of the possible choices available to Individual A. For each of the choices, please indicate whether you believe choosing that option is very socially inappropriate, somewhat socially inappropriate, somewhat socially appropriate, or very socially appropriate. To indicate your response, please place an ``x'' inside one box for each row.

History treatment instruction

The situation you are to consider is the final round of the experiment, after 19 rounds have already occurred. The table below shows you what has happened on the previous 19 rounds. Individual A now has to make a decision how many tokens to contribute on the 20th round of the experiment.

Round	Person A	Person B	Person C	Person D
Contributions, round 1	1	0	7	0
Deduction points received	0	0	0	0
Contributions, round 2	1	0	0	0
Deduction points received	0	0	0	0
Contributions, round 3	0	0	0	0
Deduction points received	0	0	0	0
Contributions, round 4	0	0	0	0
Deduction points received	0	0	0	0
Contributions, round 5	0	0	0	0
Deduction points received	0	0	0	0
Contributions, round 6	0	0	0	0
Deduction points received	0	0	0	0
Contributions, round 7	0	0	0	0
Deduction points received	0	0	0	0
Contributions, round 8	0	0	0	0
Deduction points received	0	0	0	0
Contributions, round 9	0	0	0	0
Deduction points received	0	0	0	1
Contributions, round 10	0	0	0	0
Deduction points received	0	0	0	0
Contributions, round 11	0	0	1	0
Deduction points received	0	0	0	0
Contributions, round 12	1	0	0	0
Deduction points received	0	0	0	0
Contributions, round 13	0	0	1	0
Deduction points received	0	0	0	0
Contributions, round 14	0	0	0	0
Deduction points received	0	0	0	0
Contributions, round 15	0	0	0	0
Deduction points received	0	0	0	0
Contributions, round 16	0	0	0	0
Deduction points received	0	0	0	0
Contributions, round 17	0	0	0	0
Deduction points received	0	0	0	0
Contributions, round 18	0	0	0	0
Deduction points received	0	0	0	0
Contributions, round 19	0	0	0	0
Deduction points received	0	0	0	0

Supplementary Note 3: Pre-experiment Quiz Questions

The following questions were administered to all subjects, in both experiments, prior to recording their experimental responses, as a check that they understood the rules.

1. Each group member has an endowment of 30 tokens. Suppose nobody (including you) contributes any tokens to the project. What is:

Your first stage income?

The first stage income of the other group members?

2. Each group member has an endowment of 30 tokens. Suppose you contribute 20 tokens to the project. All other group members each contribute 20 tokens to the project. What is:

Your first stage income?

The first stage income of the other group members?

3. Each group member has an endowment of 30 tokens. Suppose the other three group members contribute a total of 30 tokens to the project.

What is your first stage income if you contribute 2 tokens to the project?

What is your first stage income if you contribute 14 tokens to the project?

4. Each group member has an endowment of 30 tokens. Suppose you contribute 8 tokens to the project.

What is your first stage income if the other group members together contribute a total of 4 tokens to the project?

What is you first stage income if the other group members together contribute a total of 24 tokens to the project?

5. Suppose at the second stage you asign the following deduction points to your three other group members: 5, 0, 0.

What are your total costs of assigning deduction points?

6. What are your costs if you assign a total of 0 deduction points?

7. By how many points will your income from the first stage be reduced if you receive a total of 0 deduction points from the other group members?

8. Suppose the first stage has finished. By how many points will your income from the first stage be reduced if you receive a total of 4 deduction points from the other group members?

9. Suppose the first stage has finished. By how many points will your income from the first stage be reduced if you receive a total of 6 deduction points from the other group members?

Supplementary Note 4: Post-experiment 1 Survey Questions

Survey text follows below:

Survey Questions

Thank you for participating in the experiment on decision-making. In the following set of questions we will ask you to explain some of your actions in the last period. For answering all the questions on the survey, you will earn an additional $4 (100 points).

1. You contributed X points in the first stage of the final period. How much was the appropriate amount for you to contribute to the project (0–20)?
2. In the first stage of the last period, how much of their starting endowment did you expect each of the other three group members to contribute to the project? (0–20)
3. In the first stage of the experiment, how much was the appropriate amount for each of the other three group members to have contributed to the project?
4. You assigned Y deduction points in the second stage of the last period. Did your view about how many deduction points it was appropriate to assign change in the course of the experiment? (Y/N)
5. What was the main reason that you deducted points from the other members in your group?
6. What do you think is the main reason that others may have deducted points from you?

General Survey – Please answer the following questions:

7. Sex (M/F)
8. Age (in years, on last birthday)

9. Current study year (1st, 2nd, 3rd, 4th/honours, Master's, PhD, Graduate student, Not studying)
10. Subject of study (Economics, Other business, Psychology, Sciences, Engineering, Other, Not applicable)
11. Residency status in Australia (Australian citizen, Australian permanent resident/New Zealand citizen, New Zealand permanent resident, Student visa, Other)
12. If you are not an Australian/New Zealand citizen, your country of citizenship?
13. Your country of birth?
14. How long have you been in Australia (years)?
15. In which country did you live in most of your life?
16. Religion (Christian, Buddhist, Muslim, Hindu, Other, None)
17. How important do you consider religion in your daily life? (Very important, Rather important, Not very important, Not at all important)
18. Relationship status (Single, not in a relationship; Single, in a casual relationship; Single, in a serious relationship; Married; Divorced)
19. Do you have children (Y/N)
20. Are you employed (full or part-time) in Australia? (Y/N)
21. How many economics experiments have you participated in before this one? (None, 1–2 previous, 3–5 previous, More than 5 previous)
22. Generally speaking, would you say that most people can be trusted or that you need to be very careful in dealing with people? (Choose one answer)
    a. Most people can be trusted
    b. Need to be very careful

23. Please indicate whether you trust people from this group completely, somewhat, not very much or not at all: [Tennyson, each option should have a box on a scale of: Trust completely, Trust Somewhat, Do Not Trust Very Much, Do not Trust at all]

    a. Your family
    b. Your neighborhood
    c. People you know personally
    d. People you meet for the first time
    e. People of another religion
    f. People of another nationality

24. Do you think most people would try to take advantage of you if they got a chance, or would they try to be fair? [1-10 scale where 1 means that "people would try to take advantage of you," and 10 means that "people would try to be fair"]
25. How secure do you feel these days in your neighborhood? [Very secure, quite secure, not very secure, not at all secure]
26. How frequently do the following things occur in your neighborhood? [Very frequently, quite frequently, not frequently, not at all frequently]

    a. Robberies
    b. Alcohol Consumption in the Streets
    c. Police or military interference with people's private lives
    d. Racist behavior
    e. Drug sales in the street

27. Please tell me for each of the following actions whether you think it can always be justified, never be justified, or something in between [1-10 scale with 1 being "Never justifiable" and 10 being "Always justifiable"

    a. Claiming government benefits to which you are not entitled

b. Avoiding a fare on public transport

c. Stealing property

d. Cheating on taxes if you have a chance

e. Someone accepting a bribe in the course of their duties

f. Homosexuality

g. Prostitution

h. Abortion

i. Divorce

j. Sex Before Marriage

k. Suicide

l. Euthanasia

m. For a man to beat his wife

n. Parents beating children

o. Violence against other people

Supplementary Note 5: Coding Instructions for Survey Questions 5 and 6

A research assistant was hired to code the answers to survey questions 5 and 6. Instructions were as follows:

INSTRUCTIONS: You are about to read a number of answers given by experimental subjects in an economics experiment. Subjects in this experiment were able to assign "deduction points" to each other, resulting in a reduction of the earnings of the person who received the points. Subjects in this experiment also had the opportunity to make "contributions" which benefited other players.

The subjects were asked for what reasons they or others might have assigned deduction points. Your job is to categorise the answers, based on what sorts of reasons the respondents gave. For each possible reason, mark "1" in the corresponding cell if the subject did mention it, and "0" if the subject did not mention it. Sometimes it may be difficult to tell whether a subject has mentioned a reason or not. In this case, simply make your best guess.

The two questions which subjects answered were:

> *Question A: What was the main reason that you deducted points from the other members in your group?*
>
> *Question B: What do you think is the main reason that others may have deducted points from you?*

For both of these questions, we will be asking you,

Did the respondent endorse any of the following as reasons for their deducting point (1 = yes, 0 = no):

- Spite
- Fairness
- To encourage higher contributions
- Personal benefit
- To discourage low contributions (*this category was merged with "encourage higher contributions" for the purposes of analysis, at the request of a referee. It made negligible difference to the results.)
- To discourage high contributions
- Other

Supplementary Note 6: Responses to qualitative survey questions

Below we report the responses given to the following two qualitative survey questions:

5. What was the main reason that you deducted points from the other members in your group?
6. What do you think is the main reason that others may have deducted points from you?

Treatment P25, Question 5 (first person punishment)

- They wanted to reap benefits (money) at the cost of others' contributions. It's not fair.
- Is the fisrt period I deducted points because their contribution was not enough.
- They were contributing too low number of tokens for the project
- I did not deduct any points from other members in my group at any stage.
- when some of them contributed too little, like when all the others contributed 15 tokens and he only contributed 10.
- To reduce their income.
- I didn't deduct any points because I thought it would engender an attitude of spitefulness
- To reduce the income
- Someone distributed too little or even zero distribution which was extremely unfair to me and affected the total profit; therefore, I gave the deducted points.
- For giving a contribution cost which is lower than mine
- I only deducted one point on one occassion, when a player contributed 5 points less than everyone else. Considering the outcomes of the prior periods I thought this was a selfish move to earn a bit more money in that period, so I gave them one deuduction point as a warning!
- try to make them contribute more next time
- I never deducted points from the others
- As long as they give lower than what I give, I deduct 1 point from each of them.
- I did not
- To tell me to key in the same amount. If four of us key in the same amount, then only we can maximize our benifits.

- the lower contribution can give us higher benefit.
- Someone's contribution lower than others and I account for large part of contribution. Or someone always provide the lowest contribution.
- Make my benefits maxium
- i was confused with the rules of the experiment. I dont know when i deduct the point of my group members at the same time my own point will be deducted as well.
- For being the lowest contributing group member.
- As they did not contribute enough in comparison to the others
- In order to show the other member who was contributing less than us that he is out of line. However, I believe that not assigning any deduction points may have been more profitable in the long run (especially since there is chance someone else may assign deduction points to the undercontributor).
- That person tried to get bemefit fot himself, not care about the lost of the others in group
- 1. They contributed too little to the project 2. They dont match the average contribution of each participant 3. They contributed too high, bringing the mean contribution too high, therefore deducting them may let them feel a sense of trying to lose lesser if they continue contributing large but receiving low income.
- The one member donated 0 points to the project when we had gotten into a good rhythym of donating around 4-6 points to the pool.
- In order to promote increase in the tokens they input or to make sure that the lowest amount inputted will not go below than expected.
- Since they have just contributed very little in the experiment.
- To discourage low token contributions to the total pool.
- because of the point
- They did not contributed with tokens to the project, or they contributed with a very low amount.
- Because they contributed the lowest in the group.
- to be fair
- They didn't contribute to the project and it's unfair for the others.
- When they did not contribute any token to the project.
- They have minimal contributions points
- for going too low
- they did not contribute enough!

- I have not deducted any points from others because there's no reason at all to do it. By doing the deduction, my point would be reduced and other members' points would be reduced to. It did not make sense when none got the profit from this action.
- They did not contribute for the first few periods.
- Firstly I wanna make a test case to other members. But it seems, no one contributed in the project, so, there is no pint for me to reduce it. It seems that the other member do not relally want to play or to joint the project.
- They did not contribute at all.
- I never deducted points from my other team members. It is rude and makes no sense financially.
- To punish them for not giving the correct amount of tokens to the project.
- want them to give money in the next round
- make them contribute equa to other members.
- THE MAIN REASON WAS THAT I FELT IT WAS WRONG TO ENJOY GETING GAINS FROM THE MONEY OTHERS PUT INTO THE GROUP WHILE THE INDIVIDUAL CONTRDUTED NOTHING AT ALL
- Allocated too few points to the project
- it is because i think they have contributed too little and just wanna get the beenfits from other people's contributions.
- I did not deduct points from the other members.
- The tokens they contribute is the same as me or less than me.
- They didn't realise how to best perform the experiment
- One of the reason being that I suspect that member is the person that deduct my point
- Because they did not contribute money for the project but they still earn profit from other group members
- I do not favour those members with higher points than mine in Stage 1 so I would like to impose the deduction as the penality

Treatment P25, Question 6 (second person punishment)

- They felt I was not paying my share of the points. ie my point donation was lower than other members'
- I have no idea, since in the first period, I was the one that contributed with more points for the project. Honestly, I tought I would not be deducted any points.

- Sometimes the value of tokens I allocated to the project was lower than what they had allocated thus they deducted points from me
- It may be because they felt that I did not contribute the expected amount (i.e. they contributed more than me), or for some other reason.
- when I contributed less than their contribution
- To reduce my income.
- Because they wanted to bring me into line when I didn't contribute enough to the points.
- To increase thier income
- Maybe for the reason that my distribution is not as much as them.
- For the same reason, i.e. if I contributed fewer than them
- I think I was unfairly given deduction points several times, even when I contributed the most of all the players. I think at least one person in the group didn't understand the instructions and thought they reaped a benefit from deducting points from other players.
- I contributed less than anyone else
- Because I didn't contribute as much as they wished
- Because the amount I give is lower than what they gave.
- Because I did not play fair
- They don't understand the rules. They thought the one who contributed the most will earn the most. So they deduct my points if i am the one who contribute the most which is FALSE!!!!!!!!!!!!!!!!!!!!!
- i input more tokens in contribution.
- I gained most in the first stage.Also they afraid to be deducted points from others members. In order to be fair , they want four members win almost the same amount.
- because I didn't contributed enough points to the project and this makes my earning higher than others, they want to decrease my benefits to achieve a balance.
- They dont know the rules like me, OR they are willing to get less so that other group members get LESSER.
- I think it was usually for being the lowest contributing group member other than me. However, this theory failed in one case where I contributed the highest number of tokens and was given three deduction points, causing a great deal of confusion from me and a lashing out at the team members the next round, which ended up backfiring.
- Due to contributing only little
- For contributing less than them.

- They thought I contributed too little
- For the 20 periods, they only deducted me if I contributed below the average
- because I chose to only put in 0 when they had put tokens in. I was hoping that everyone would start putting in 0 but obviously when they received deduction points for the first time they got scared :(shame really I was hoping by around round 5 that everyone would be on team 0
- Below their expected input of tokens
- Since I did not contriute much in the experiment
- Below expected token contributions to the total pool.
- higher point
- Because they thought I was not contributing with enough tokens to the project.
- Because i have contibuted the lowest in the group.
- because i didnt contribute anything
- I didn't contribute to the project.
- I did not contribute any token at one of the periods and that's when I got the deduction.
- I have the least contributions points
- for going too low
- because i did not contributed the expected asmount (too little).
- For the first period, I thought they did not understand the rule or they did not like my project contribution on that period.
- Randomly deducted my points. Not sure.
- No reason, no one contributed, may be just a little, but most the time their contribution is 0 as well as the deducted pints. They don want to play nor contribute to the project.
- I did not get any points deducted.
- They either got bored or were trying to provoke other team members to invest some of their points.
- To punish me for not giivng enough tokens to the project
- because they gave money but I didn't
- I have no ideal. even when i contribute highest. they still deduce my point.. @@. may be they deduce for fun.
- SOMETIMES I FELTTHAT THAT THE OTHER MEMBERS OF THE GROUP GAVE ME DEDUCTION POINTS TO PREVENT ME FROM GETTING MORE TOKENS. ALSO THE ABOVE REASON FOR THE PREVOIUS QUESTION COULD ALSO BE APPLIED

- They had points deducted from them. The contribution from a person was too little relative to the rest of the other people.
- i think they didnt think much, which i also only have been deducted for the first peiord only in thed 20 rounds.
- After seeing the contribution, they might have felt cheated.
- The difference of the tokens contributed by me and others is big
- They don't understand how to maximise their money' poor decision skills
- Not really sure. Probably, they just want to make it more interesting rather than playing stagnant for the whole period
- They want to punish me
- Some may have extra points to do so because they have more points than me.

Treatment P20, Question 5 (first person punishment)

- If they were being obviously selfish and contributing signifiacntely lower than the rest of the group.
- I did not deduct any points from other members, because it cost me points and I didn't want them to lose money either.
- As “punishment” for not contributing enough
- I see no points in deducting points from other group members. Also, doing so may create further risk from their contribution, that may do harm to the group benefit.
- Because they were not contributing. So I guess deducting others point is the only way to communicate and to motivate.
- because he gave 0 points when all of us gave 5 points
- I did not decduct points from the other members
- I did not deduct any points from the other members in my group.
- Becasue they should contribute, even in a small amount.
- I DID NOT DEDUCT ANY
- Only if they contributed a lot less than me generally. This changed as I decided to slowly reduce my contribution to 0 (most efficient for all group members)
- They contributed none or little tokens compared to others
- Because I contributed points but they didn`t so I felt it was unfair.
- For not contributing enough to the project - I would typically deduct points from members who only contributed 1 or 2 points to the project.

- let them reduce their contribution
- If they contributed a smaller amount then me and the median of the group, they deserve that deduction.
- I didn't want to cost any point on this.
- I did not deduct points, so no comment
- I would look for overall contribitom, if any outliers were present at the lower end of the scale then a deduciton of 1 point would occur. Those who contributed 0 got 2 deductions. It must be fair contribution by each member and not a selfish act, thus those who gave very less compared to the others (>2) were penalised by deduciton points.
- I did not deduct any points from the others from the start because I felt that if everyone does not deduct points at all it would be the greatest benefit for everyone.
- I have not deducted any points off the other members. Since i am looking towards maximizing my profit, the best way is to not use my points to deduct others' points. At the same time, make sure that if others are deducting my points, they are not deducting more than amount i earned from their group contribution. If the consistently deduct my points for not donating to group, i will consider donating some to avoid point loss from their deduction.
- The less they contributed to the project the more deduction point I gave them.
- If they didn't contribute enough to the project, I would deduct a point or two from them to remind them to contribute more next period. Usually when I did this, it was effective
- they do not contribute for the same amount. not evenly
- Because they deducted points from me as well when i decided not to deduct points from them at first
- I only deducted points when the other members contributed less than me.
- When they deduct my point for no reason.
- It is because this member invest less than others
- I didn't, because they cost.
- Contributing less than what is expected
- It is to hint them that they provide the wrong answer and average the marks with other group members
- I think that the best way was not to contribute any points to the group. However, we didn't make an agreement amd i wanted to express my feelings.
- Being unreasonable which is either contributing too much or too low.

- I did not deduct points from the other group members, 1. Because its mean to take other's money from them to higher your own earnings, and 2. It would have cost both me and the member so it did not make financial sense
- To get them to contribute more points in following rounds. If I deduct too much they might get emotional. If I don't deduct at all they will keep trying to undercut me.
- The money they contribute is less than the average contributed money of the group
- some group member contritube too little ammount and its not good for the total contribution, for fair, i think i should deduct this group member's point.
- contribute less
- To push the investment amount of group members higher
- to increase my point
- i want to decrease their income
- I did not
- For fun. Didn't cost a lot to make others lose money
- No i didnt deduct any points from the other members, simply because they did not deduct mine. Also because deducting their points will mean sacrificing my own points, not beneficial to both parties.
- free ride
- To have “fairer” returns
- they contributed les to the project than other memebers
- I didnt at all because it was in no way in my best interest because then i would have lost points as well
- to decrease income
- I didnt deducted!
- I want every members get equal points.
- Not to avoid losses for me and him/her, send them 0
- they contributed less than me
- yolo
- I was mad at them
- none
- i did not do such a thing
- Because they kept putting money into the kitty when it was only detrimental for themselves

- i didnt, i didnt feel any need to deduct any points

Treatment P20, Question 6 (second person punishment)

- They didn't deduct any from me- but I would say if they did similar reasons to previous question.
- Because I contributed less tokens to the project.
- As "punishment" for not contributing enough
- I think other group members think the same like me; that is, there is no point in deducting points from others, because it does no good to them as well as others.
- They didnt. Guess if they did maybe because Im no longer contributing.
- there was no such ocassion as i matched all their contributions. However, they would have done so if they gave something and i gave considerably lesser
- Just for understanding how the system works in the first periods
- In the first round, I was the only one who did not contribute to the project (the three others contributed 5 points each). Therefore, their total income was slightly lower because I did not contribute and hence someone in my group tried to “penalize” me for doing so.
- The feeling of unbalance.
- BECAUSE I DID NOT CONTRIBUTE TO THE PROJECT BUT I EARN FROM WHAT THEY CONTRIBUTED.
- No one ever did. I was careful not to reduce my contribution significantly
- The amount of tokens I contributed was far more less compared to the others.
- They thought that I contributed too few points gor the group.
- I always contributed the most out of any other member of the group - so maybe they were trying to downsize my profit?
- I did the smallest contribution
- Nobody deducted points from me. However, it is fair to assume if they did it is because I did not contribute enough to the pile.
- They didn't want to lose any point on this.
- No one dedcuted points from me. I guess people were just feeling kind today
- Possibly because they beleive that I have deducted points from them for 3-4 periods as that person had only contributed 0-1 points whilst everyone else contributed 3, thus to get back at us and to decrease our earnings to match theirs this member has decided to take such action to deduct points from me.

- I think that the main reason would be that they have not grasp the best idea to benefit everyone including themselves. I could even guess which member might have did the deduction on me but I did not deduct any points at all just to be safe and deduction of points does not benefit anyone including myself anyways.
- Initially, they deduct my point because i am not contributing to group. Eventually, they realise that the best way to maximise indiviual profit is to have the members in the whole group not donating at all and not deducting. Suggestion: It is perhaps more interesting expoperiment if the group contribution yields more income than keeping for self.
- When I contributed less to the project.
- I got deducted from a few times, and I'm not sure why. It was usually a period when I contributed enough or more than anyone else. Probably just greed was the reason.
- they think i earn more than theirs
- I am not sure
- Team members might have deducted points from em if I had contributed a lower amount to the project than they had
- They want to have higher income than me.
- It is because I contribute less to projects
- Stupidity, irrationality.
- Misidentification; trying to get me to reduce my contribution
- It is because my mark is the lowest among the all
- Compared with others, I put the least points in the group.
- 1. contributed lesser than they did 2. illogical thinking
- They may have thought my contribution was too low and my own profit too high
- Possibly the same reason. Possibly 'revenge' for the points I deducted, since I always targeted the lowest contributer if any.
- The money I contribute is less than the average contributed money of the group. For every investment, I will lose 0.05x contribution if everyone contribute the same amount.
- Contribute less than other group member
- did not contribute enough.
- Reactionary from pushing the investment higher than they were willing to go
- to decrease my point
- they want to decrease my income
- they are idiots. Lose-lose is the stupidest thing ever.

- Lack of contribution to the project. Most other group members did not contribute in the last 3/4 periods anyway
- It could be because of 0 contribution to the project, but my 3 other project partners contribute 0 from the second stage onwards, and deduct no points from each other, so I assume we had a mutual agreement not to deduct until the 20th period.
- free ride
- N/A
- They did not deduct any point from me but I believe they would have done so if I had contributred less than them in the project
- because i didnt contribute what they thought was enough to a project
- to decrese income
- Because they didn't understand that, it only makes you lose money.
- they don't want me to get more points.
- I don't know
- I contributed less than them
- they probably got mad they put a lot and i didnt put any
- they didn't, probably because I wasnt rude to begin with
- they didn't want me to earn my maximum income
- I did not recieved any deduction points
- They mistakenly thought that contributing nothing to the project was bad.
- no one did, i suppose i was genorous enough for them

Supplementary References

1. Rabin, M. Incorporating fairness into game theory and economics. *American Economic Review* **83,** 1281–1302 (1993).
2. Dufwenberg, M. & Kirchsteiger, G. A theory of sequential reciprocity. *Games and Economic Behavior* **47,** 268–298 (2004).
3. Krupka, E. L. & Weber, R. A. Identifying social norms using coordination games: Why does dictator game sharing vary? *Journal of the European Economic Association* **11,** 495–524 (2013).
4. López-Pérez, R. Aversion to norm-breaking: A model. *Games and Economic Behavior* **64,** 237–267 (2008).
